# Enrichment of *Triticum aestivum* gene annotations using ortholog cliques and gene ontologies in other plants

**DOI:** 10.1186/s12864-015-1496-2

**Published:** 2015-04-15

**Authors:** Dan Tulpan, Serge Leger, Alain Tchagang, Youlian Pan

**Affiliations:** Information and Communications Technologies, National Research Council Canada, Moncton, New Brunswick E1A 7R1 Canada; Information and Communications Technologies, National Research Council Canada, Ottawa, Ontario K1A 0R6 Canada

## Abstract

**Background:**

While the gargantuan multi-nation effort of sequencing *T. aestivum* gets close to completion, the annotation process for the vast number of wheat genes and proteins is in its infancy. Previous experimental studies carried out on model plant organisms such as *A. thaliana* and *O. sativa* provide a plethora of gene annotations that can be used as potential starting points for wheat gene annotations, proven that solid cross-species gene-to-gene and protein-to-protein correspondences are provided.

**Results:**

DNA and protein sequences and corresponding annotations for *T. aestivum* and 9 other plant species were collected from Ensembl Plants release 22 and curated. Cliques of predicted 1-to-1 orthologs were identified and an annotation enrichment model was defined based on existing gene-GO term associations and phylogenetic relationships among wheat and 9 other plant species. A total of 13 cliques of size 10 were identified, which represent putative functionally equivalent genes and proteins in the 10 plant species. Eighty-five new and more specific GO terms were associated with wheat genes in the 13 cliques of size 10, which represent a 65% increase compared with the previously 130 known GO terms. Similar expression patterns for 4 genes from Arabidopsis, barley, maize and rice in cliques of size 10 provide experimental evidence to support our model. Overall, based on clique size equal or larger than 3, our model enriched the existing gene-GO term associations for 7,838 (8%) wheat genes, of which 2,139 had no previous annotation.

**Conclusions:**

Our novel comparative genomics approach enriches existing *T. aestivum* gene annotations based on cliques of predicted 1-to-1 orthologs, phylogenetic relationships and existing gene ontologies from 9 other plant species.

**Electronic supplementary material:**

The online version of this article (doi:10.1186/s12864-015-1496-2) contains supplementary material, which is available to authorized users.

## Background

Gene orthology forms the backbone of comparative and evolutionary genomics and it represents a central piece in many computational methods for functional annotation of genes particularly relevant for newly sequenced plant species. Orthologs are genes that evolved from their last common ancestor after a speciation event [1,2] and are essentially considered to be the ‘same’ gene in different species. In comparison, paralogs are genes which are derived via a gene duplication event and although evolutionarily related, they are not the ‘same’ gene and are unlikely to have all the same function in different species. The precise identification of orthologs and paralogs is a quintessential step in comparative genomics and functional analysis of genes.

Existing orthology prediction methods can be broadly grouped into two categories [3]: (i) graph-based methods that cluster pairs of genes based on (typically protein) sequence similarity (e.g. InParanoid [4], RoundUp [5], COG [6], KOG [7], eggNOG [8], OrthoDB [9], OrthoMCL [10], OMA [11]), and (ii) tree-based methods, which cluster genes and aim for the reconciliation of the protein and the species trees (e.g. TreeFam [12], Ensembl Compara [13], PhylomeDB [14], LOFT [15]). Systematic evaluations of these methods including advantages, disadvantages, challenges and validation are discussed in the literature [16,17].

A widely adopted approach for orthology prediction is the Reciprocal Best BLAST Hit (RBBH) method (also known as ‘bidirectional best hit’) [6,18], which identifies orthologous genes between two species that are more similar to each other than to any other gene in the same species. While the RBBH method was proven to provide a solid bidirectional bridge between orthology and bidirectional best hits inferred from sequence similarity [19], it is by far not perfect given its limitations caused by evolutionary events such as gene loss and gene duplications or by incomplete genomic sequences. Such limitations lead to false positives representing incorrect labelling of paralogs as orthologs [20]. Nevertheless, in particular circumstances where trusted orthologs are required, such as information enrichment in genome annotation [19], the RBBH method generates high quality 1-to-1 orthologs, which can be further used to seed orthologous groups [20].

**Figure 1:**
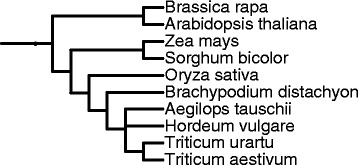
Phylogenetic tree with 10 plant species obtained with iTOL v2 [61].

While we acknowledge the potential positive contribution of paralogy relationships for gene annotation, in this work we follow a very conservative approach and thus, only 1-to-1 ortholog cliques detected in 10 plant species (Table 1) are used to enrich existing *Triticum aestivum* (bread wheat) gene annotations. Here a clique is defined as a set of genes (one in each species) that are pairwise 1-to-1 orthologs. Besides bread wheat, we selected gene annotations for 9 other plant species, which are either evolutionary close to bread wheat (Figure 1) or well annotated model organisms such as: *Aegilops tauschii* (Tausch's goat grass), *Arabidopsis thaliana* (thale cress or mouse-ear cress), *Brachypodium distachyon* (purple false brome), *Brassica rapa* (white turnip), *Hordeum vulgare* (barley), *Oryza sativa subsp. japonica* (rice), *Sorghum bicolor* (sorghum, durra, jowari, or milo), *Triticum urartu* (wild wheat) and *Zea mays* (maize)*.*

**Table 1:** **Information about ten plant species included in this study**

**Species**	**NCBI Taxonomy ID**	**Number of chromosomes**	**Estimated genome size [Mb]**	**Number of selected DNA/proteins (Ensembl Plants)**
*Aegilops tauschii*	37682	2n = 14 (DD)	4,360	33,849
*Arabidopsis thaliana*	3702	2n = 2x = 10	135	27,416
*Brachypodium distachyon*	15368	2n = 2x = 10	270	26,552
*Brassica rapa*	51351	2n = 2x = 20	529	41,018
*Hordeum vulgare*	112509	2n = 2x = 14	5,300	24,211
*Oryza sativa*	39947	2n = 2x = 24	383	35,679
*Sorghum bicolor*	4558	2n = 2x = 20	730	34,496
*Triticum aestivum*	4565	2n = 6x = 42 (AABBDD)	17,000	98,779
*Triticum urartu*	4572	2n = 14 (AA)	4,940	33,424
*Zea mays*	4577	2n = 2x = 20	2,300	38,741

To enhance the confidence in our orthology prediction, we use two types of information as base input sequences for our RBBH prediction: DNA coding sequences and their corresponding proteins. We define as 1-to-1 orthologs those pairwise orthology relations commonly predicted by the RBBH method using as input the aforementioned information. In case of disagreement between the two predictions, the putative individual orthologs are dismissed, at the expense of losing potentially valid orthologs. For example, if the coding sequence representing gene TRAES_5BL_DAE1BD995 in *Triticum aestivum* is an RBBH of the corresponding DNA for gene TRIUR3_21564 in *Triticum urartu*, and if the corresponding protein sequences associated with the same genes are also RBBHs, then we accept the pair (TRAES_5BL_DAE1BD995, TRIUR3_21564) as 1-to-1 ortholog.

A related approach where cliques of OMA [11] orthologs and paralogs were used to enhance functional annotation of prokaryotic genes was proposed by Skunca et al. (2013) [21]. Their approach assigned novel GO terms to orthologous genes based on majority voting, i.e. if a certain GO term exists in 50% or more of the genes in a clique, then it is assigned to the remaining genes in the same clique. A more recent study [22], proposes a computational framework (OrthoClust) that integrates single species co-association networks into data clusters across multiple species via orthology relationships. Their framework is applied on RNA-Seq expression profiles of *C.elegans* and *D. melanogaster* from the modENCODE consortium.

Here, we introduce a novel 3-step gene ontology enrichment model that relies on a set of cliques containing pairwise 1-to-1 predicted orthologs among multiple plant species, a set of known annotations for genes in each plant species and the phylogenetic relationship of those species. Once a target plant species such as *T. aestivum* is selected, the model calculates gene ontology scores based on the phylogenetic proximity between two species, the overall hierarchy of gene ontology and the predicted orthology relationships. Based on the calculated scores and further refinement using the augmented degree of novelty compared to existing annotations and a minimum score threshold, novel GO terms are assigned to the genes in the target species. Overall, based on clique sizes equal or larger than 3, our model contributed to the existing gene-GO term associations in *T. aestivum* by enriching 7,838 (8%) genes, of which 2,139 had no previous annotation.

## Methods

### Data sets

We downloaded DNA coding sequences, protein sequences and Gene Ontology (GO) vocabulary for functional annotation from the FTP site of Ensembl Plants version 22 [23] for 10 plant species: *Aegilops tauschii*, *Arabidopsis thaliana*, *Brachypodium distachyon*, *Brassica rapa*, *Hordeum vulgare*, *Oryza sativa*, *Sorghum bicolor*, *Triticum aestivum*, *Triticum urartu* and *Zea mays*. Coding DNA and protein sequences were pre-processed and only those corresponding to longest transcripts were selected for pairwise BLAST runs. Annotations and physical map information was acquired programmatically from the Gramene MySQL database build 41. The physical mapping information for *Aegilops tauschii* and *Triticum urartu* was complemented with information extracted and processed from the original publications of the two species [24,25].

### Orthology prediction

We implemented a Reciprocal Best BLAST Hit (RBBH) approach for 1-to-1 orthology prediction inspired from previous work applied to human and mouse genomes [26]. Two BLAST runs are executed for each pair of plant species and for each sequence type (DNA, protein) to identify reciprocal best hits (RBBHs). A 1-to-1 orthology relationship is assigned for those pairs of genes that are bidirectional hits within a confidence interval (e-value ≤ 10^−5^).

### Cliques of orthologs discovery in ten plant species

Cliquer version 1.21[27] was used to identify cliques of orthologs in 10 plant species. Cliquer is a highly efficient graph-based algorithm for finding cliques in an arbitrary weighted graph. It uses an exact branch-and-bound algorithm developed by Patric Östergård at Aalto University in Finland. Custom scripts were used to convert pairwise orthology data into DIMACS-formatted files required by Cliquer. Each gene represents a node in the graph and each edge represents an orthologous relationship. All edges in the graph were equally scored (score = 1). Cliquer was executed using the following parameters: −*a*, −*x* and *–m* 3. Cliquer generated all cliques with size between 3 and 10 using as input 45 files with unique pairs of orthologs between the 10 plant species in less than one hour (~44 minutes) on a desktop computer running Linux Ubuntu (64-bit) kernel 3.8.0-35-generic, with 256 GB of RAM (less than 2GB used) and two 16-core CPUs (no parallelism used).

**Figure 2:**
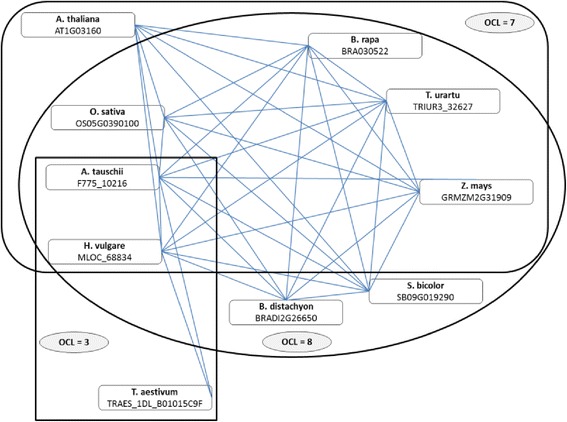
Three cliques with OCLs equal to 3, 7 and 8.

Orthologs for each pair of species are considered as input. We introduce the notion of “O*rtholog Clique Level*” (OCL) for a given gene representing the size of the clique where it resides. The OCL also represents the number of species among which the gene has 1-to-1 orthologs including the host species. Figure 2 represents 10 genes in 10 plant species whose orthologs form a graph with 3 cliques with OCLs equal to 3, 7 and 8.

### Orthologs clique validation using overlapping gene ontologies

We use the Gene Ontology (GO) vocabulary for functional annotation provided in Ensembl Plants for the 10 plant species to calculate the percentage of overlapping GO terms for all genes belonging to a clique of size *k*. An overlap score is calculated as follows:

1 $$ GO\  Set\  Overlap(C)=\frac{\left|{\cap}_{i\kern0.3em =\kern0.3em 1}^k{S}_i\right|}{{ \min}_{i=1:k}\left|{S}_i\right|}*100 $$

where *C* is a clique with OCL = *k* (here *k* is between 3 and 10), *S*_*i*_ is a set of unique GO terms associated with a gene *i* from clique *C*. The GO set overlap percentage is higher when large numbers of GO terms associated with each gene in a clique are common to all the genes. For example, assume a clique with OCL = 3 contains the following genes with corresponding GO terms listed in parentheses: AT2G02170 (GO:0005886, GO:0008150), OS02G0116800 (GO:0005886,GO:0008150) and SB04G001240 (GO:0005886, GO:0008150, GO:0003677). The GO set overlap percentage is 100%, since two GO terms (GO:0005886, GO:0008150) occur in all three genes and the smallest set of GO terms associated to a gene has size 2 for the Arabidopsis and rice genes.

For comparison purposes, to distinguish between well predicted cliques of orthologs and randomly predicted ones, we generated a set of cliques with OCLs between 3 and 10 populated with genes selected uniformly at random from complete pools of genes for each of the 10 plant species. For each clique size (OCL), we generated as described above an equal number of cliques and we calculated the *GO Set Overlap* as described by Equation 1.

### Gene ontology enrichment

**Figure 3:**
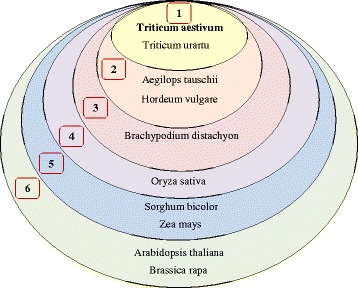
Evolutionary proximity grouping of 10 plant species centered around bread wheat. Each group has a score ranging from 1 to 6, which is used for assignment of GO terms to bread wheat genes.

We use the same Gene Ontology (GO) vocabulary for functional annotation provided in Ensembl Plants to seed and enrich the functional annotation of *Triticum aestivum* genes. We propose a scoring function for GO term assignment to wheat genes based on the knowledge of evolutionary proximity between *Triticum aestivum* and the other 9 plant species (Equation 2). Based on the phylogenetic representation of the 10 plant species (Figure 1) we divide them into 6 groups and assign scores, *G*_*score*_ (*i*), from 1 to 6 to each species *i* (Figure 3). Then we calculate the score for each GO term *go* that belongs to a clique *c*, which contains a wheat gene as follows:

2 $$ G{O}_{score}\left(\mathit{\mathsf{g}}o,c\right)=\kern0.5em {\sum}_{i=1}^{\left|c\right|}\delta \left(\mathit{\mathsf{g}}o,i\right)*\frac{1}{G_{score}(i)} $$

where *|c|* is the size of clique *c* (i.e. the number of orthologous genes in clique *c*),

$$ \begin{array}{l}\delta \left( go,i\right)=\left\{\begin{array}{cc}\hfill 0\hfill & \hfill \mathrm{if}\  go\ \mathrm{is}\ \mathrm{not}\ \mathrm{a}\ \mathrm{GO}\ \mathrm{t}\mathrm{erm}\ \mathrm{o}\mathrm{f}\ \mathrm{gene}\ \mathrm{corresponding}\ \mathrm{t}\mathrm{o}\ \mathrm{species}\ i\hfill \\ {}\hfill 1\hfill & \hfill otherwise\hfill \end{array}\right.\\ {}\mathrm{a}\mathrm{nd}\ {G}_{score}(i)=\left\{\begin{array}{l}\begin{array}{l}\begin{array}{l}\begin{array}{lll}1\hfill &, & if\  species\ i\ \in \left\{T. aestivum,T. urartu\right\}\hfill \end{array}\hfill \\ {}\begin{array}{lll}2\hfill &, & if\  species\ i\ \in \left\{A. tauschii,H. vulgare\right\}\hfill \end{array}\hfill \end{array}\hfill \\ {}\begin{array}{lll}3\hfill &, & \hfill if\  species\ i\ \in \left\{B. distachyon\right\}\kern4em \hfill \end{array}\hfill \end{array}\hfill \\ {}\begin{array}{l}\begin{array}{ccc}4\hfill &, & if\  species\ i\ \in \left\{O. sativa\right\}\hfill \end{array}\hfill \\ {}\hfill \begin{array}{c}\hfill \begin{array}{lll}5\hfill &, & if\  species\ i\ \in \left\{S. bicolor,Z. mays\right\}\hfill \end{array}\hfill \\ {}\begin{array}{lll}6\hfill &, & if\  species\ i\ \in \left\{A. thaliana,B. rapa\right\}\hfill \end{array}\hfill \end{array}\hfill \end{array}\hfill \end{array}\right.\kern1em .\end{array} $$

For the 10 plant species considered in this study, the maximum *GO*_*score*_ is 4.32, which corresponds to a GO term present in all genes in a clique of size 10 (1 + 1 + ½ + ½ + 1/3 + ¼ + 1/5 + 1/5 + 1/6 + 1/6 = 4.3166667 ≈ 4.32).

We use a score threshold of *G*_*T*_ = 0.5 above which we assign GO terms to a wheat gene. The choice of 0.5 for the threshold value is rooted in the significant phylogenetic proximity of *Triticum aestivum* to other closely related cereals such as *Triticum urartu*, *Aegilops tauschii* and *Hordeum vulgare*, all being part of groups with scores equal to 1 and 2. A GO term assigned to a gene in a species with group score equal to 2, will contribute 1/2, i.e. 0.5 to the overall *GO*_*score*_, thus being considered sufficiently significant to be assigned to the orthologous wheat gene.

### AgriGO analysis

The following parameter settings were used in the AgriGO [28] analysis: (i) Fisher’s exact test with Benjamini-Yekutieli (FDR under dependency) multiple comparison correction and (ii) significance level α = 0.05.

## Results and discussion

### 1-to-1 ortholog cliques

For every pair of plant species we predicted 1-to-1 orthologs using the RBBH method. Additional file 1: Table S1 provides details with respect to the total number of 1-to-1 orthologs predicted when DNA and protein sequences were used. Based on these results we considered the intersection of the DNA and protein orthology predictions for further exploration. We found cliques of 1-to-1 orthologs among the 10 plant species and grouped them based on the Ortholog Clique Level (OCL), which represents the clique size. Given the sparsity of gene ontologies in plants, we focused a large part of our analyses on ortholog cliques of size 10 while we provide additional information regarding different aspects of genes in cliques with OCLs between 3 and 10 [see Additional file 2: Figures S1 and Figure S2]. Each ortholog clique consists of genes connected to every other orthologous gene and thus they are expected to have similar functions.

**Figure 4:**
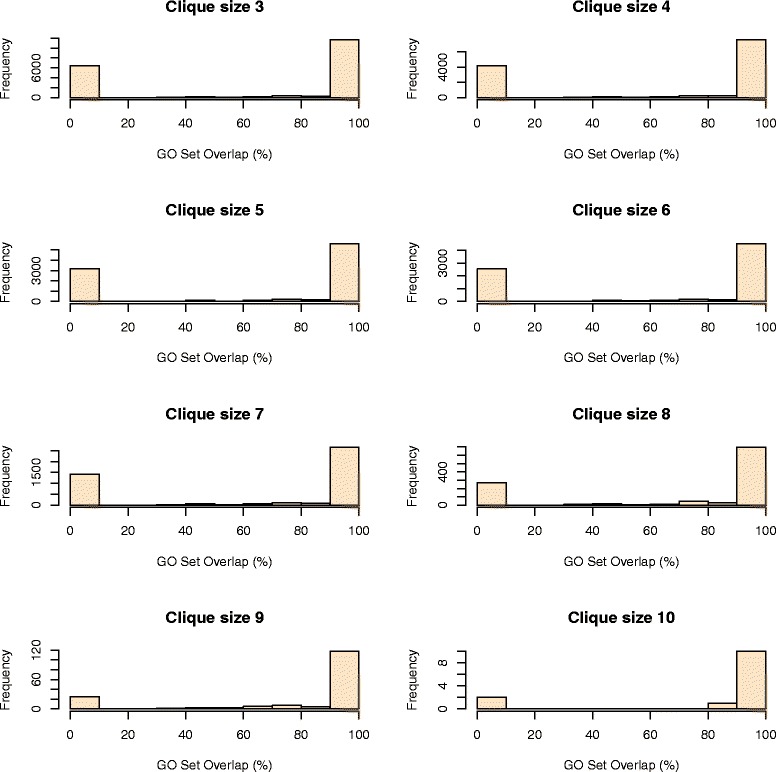
Histograms representing the GO Set Overlap for cliques of size 3–10. Frequencies of cliques with GO Set Overlap percentages within a given interval.

**Figure 5:**
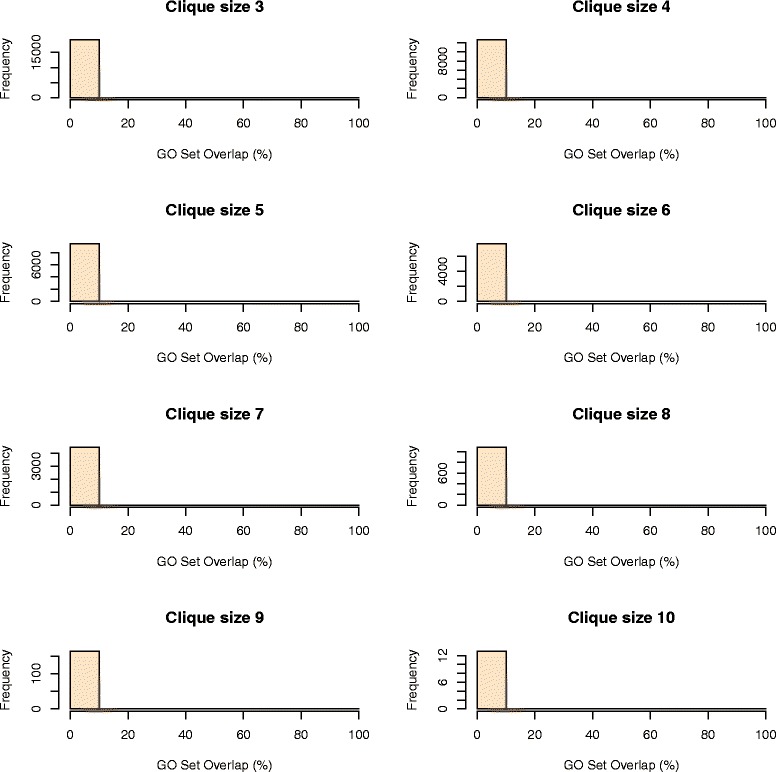
Histograms representing the GO Set Overlap for randomly generated cliques of size 3–10. Frequencies of random cliques with GO Set Overlap percentages within a given interval.

Here we provide evidence that our cliques of orthologs are well defined and validated by looking at the overlap of GO terms already assigned to genes in the 10 plant species. Using the scoring formula proposed in Equation 1, the vast majority of genes in cliques with OCLs between 3 and 10 have a *GO Set Overlap* equal to 100%. This means that the GO terms assigned to the least annotated gene in a clique occur in all the other annotations of genes from the same clique (Figure 4), which validate the pairwise orthology predictions for that clique. In contrast, if the orthology predictions would be invalid, then the sets of GO terms assigned to genes in the same clique will not overlap, thus leading to a *GO Set Overlap* equal to 0%. Figure 5 depicts *GO Set Overlap* percentages for an equal number of cliques with OCLs between 3 and 10 populated with genes assigned uniformly at random from the complete set of genes in each of the 10 plant species. As expected, for randomly generated cliques of orthologs, the vast majority of GO Set Overlap percentages equals zero.

### GO scores and OCLs

**Figure 6:**
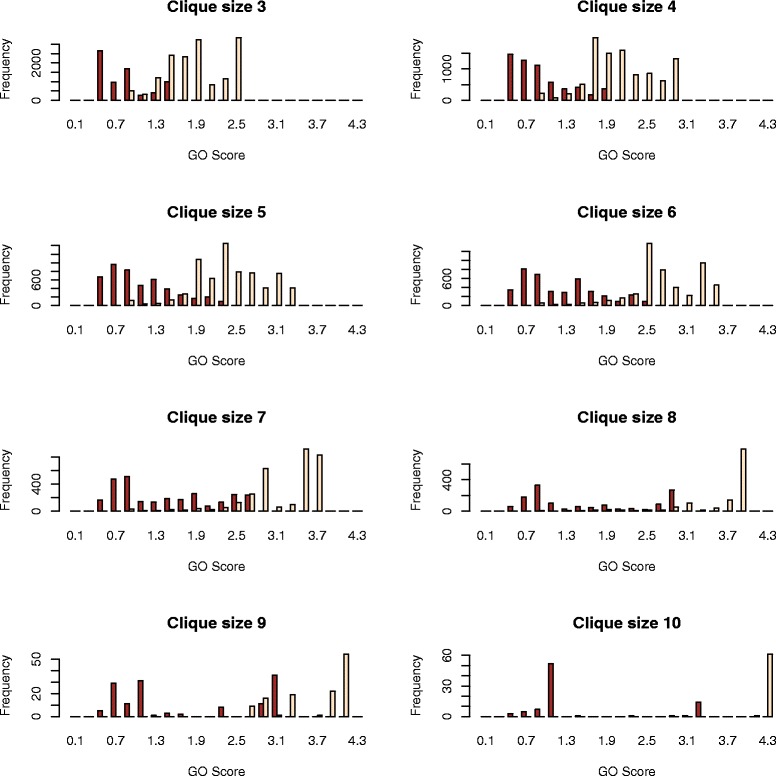
Histograms representing the frequency of GO scores for cliques of size 3–10. Frequencies of GO scores for new GO terms are represented with brown bars and those for original GO terms are displayed with bisque bars.

The GO score is tightly connected with OCLs via orthology relationships. Figure 6 depicts the frequency of original and newly assigned GO terms for all GO scores corresponding to cliques with OCLs between 3 and 10. It can be observed that a GO score threshold of 0.5 provides a minimum significant cut-off above which the majority of GO terms can be considered significant for all OCLs. The GO scores for original GO terms are consistently higher than those assigned to novel terms. Since each original GO term is already part of the gene ontologies associated to wheat genes, their corresponding GO score given by Equation 2 receives a +1 contribution factor due to wheat being a member of the evolutionary proximity group 1 (Figure 3).

### Characterization of genes in cliques of size 10

Based on our analysis, we discovered 13 cliques of size 10 (Table 2), each consisting of a set of 10 genes (one from each species) that are pairwise 1-to-1 orthologs. To further validate the correctness of the 1-to-1 orthology predictions, we explored the annotation of genes in cliques of size 10 [see Additional file 1: Table S2 ] using information from the following public databases: Ensembl Plants release 22/Gramene release 41[23], Phytozome 9.1 [29], NCBI, MIPS Barley Genome DB (IBSC) [30], RAP-DB [31] and AraMemnon [32]. With the exception of *T. aestivum* genes, which are mostly un-annotated in all databases, the majority of the genes in the other 9 species have annotations available. *B. distachyon* is an exception where 5 genes (*BRADI3G16010*, *BRADI3G16010*, *BRADI2G44260*, *BRADI4G15010* and *BRADI3G42580*) are completely un-annotated in all databases. The available annotations are almost identical for 1-to-1 orthologous genes in each clique of size 10 [see Additional file 1: Table S2 ].

**Table 2:** **13 cliques of size 10 with 1-to-1 orthologs in all 10 plant species**

**Clique**	**ATA**	**ATH**	**BDI**	**BRA**	**HVU**	**OSA**	**SBI**	**TAE**	**TUR**	**ZMA**
1	F775_06552	AT3G44600	BRADI3G42750	BRA019430	MLOC_38535	OS08G0557500	SB07G024330	TRAES_7DS_8020BEEC2	TRIUR3_22015	GRMZM2G049525
2	F775_11186	AT1G30010	BRADI1G39140	BRA032352	MLOC_55696	OS12G0407300	SB01G044780	TRAES_7DL_659883F3D	TRIUR3_27785	GRMZM2G023983
3	F775_08951	AT2G47910	BRADI3G16010	BRA021447	MLOC_8265	OS08G0167500	SB02G024420	TRAES_6AS_87906149C	TRIUR3_09163	GRMZM2G106164
4	F775_06961	AT3G13220	BRADI1G36410	BRA039378	MLOC_66857	OS06G0607700	SB10G023750	TRAES_7DL_439CC6EA0	TRIUR3_24106	GRMZM2G076526
5	F775_11259	AT2G21070	BRADI3G01970	BRA030323	MLOC_14151	OS02G0121200	SB04G001730	TRAES_6AS_AD173C5A3	TRIUR3_05931	GRMZM2G090156
6	F775_11739	AT2G40760	BRADI2G44260	BRA016973	MLOC_63819	OS05G0323100	SB09G011890	TRAES_6AS_FD8F6B539	TRIUR3_17179	GRMZM2G087671
7	F775_31011	AT3G55360	BRADI2G03297	BRA007154	MLOC_59964	OS01G0150000	SB03G006070	TRAES_3B_90F2B79E9	TRIUR3_25686	GRMZM2G481843
8	F775_27767	AT3G55760	BRADI4G15010	BRA023783	MLOC_9792	OS11G0586300	SB05G022830	TRAES_4BS_2159A428F	TRIUR3_31794	GRMZM2G069092
9	F775_13173	AT5G06550	BRADI4G16020	BRA009205	MLOC_65909	OS11G0572800	SB05G022250	TRAES_7DL_96FFFB41E	TRIUR3_19243	GRMZM2G078198
10	F775_13768	AT1G63660	BRADI3G20590	BRA027795	MLOC_34318	OS08G0326600	SB06G033930	TRAES_6AS_E6DEE586C	TRIUR3_04203	GRMZM2G136283
11	F775_08440	AT4G35870	BRADI1G75920	BRA010507	MLOC_68300	OS03G0137400	SB01G047810	TRAES_4DL_11B05CF85	TRIUR3_30140	GRMZM2G059891
12	F775_30997	AT4G35250	BRADI3G42580	BRA020809	MLOC_3618	OS08G0553800	SB07G024590	TRAES_7DS_E3B38CA36	TRIUR3_18085	GRMZM2G143917
13	F775_08503	AT1G03190	BRADI2G36360	BRA030524	MLOC_66388	OS05G0144800	SB09G003450	TRAES_1AS_A25EED9EA	TRIUR3_25030	GRMZM2G097605

The 1-to-1 orthologs included in these cliques are RBBHs predicted using as input both DNA and protein sequences. **ATA** = ***A*** *egilops* ***ta*** *uschii*, **ATH** = ***A*** *rabidopsis* ***th*** *aliana*, **BDI** = ***B*** *rachypodium* ***di*** *stachion*, **BRA** = ***B*** *rassica* ***ra*** *pa*, **HVU** = ***H*** *ordeum* ***vu*** *lgare*, **OSA** = ***O*** *ryza* ***sa*** *tiva*, **SBI** = ***S*** *orghum* ***bi*** *color*, **TAE** = ***T*** *riticum* ***ae*** *stivum*, **TUR** = ***T*** *riticum* ***ur*** *artu*, **ZMA** = ***Z*** *ea* ***ma*** *ys*.

**Clique 1** consists of a set of 10 pairwise 1-to-1 orthologs in 10 plant species potentially representing *cyclophilin71*, a member of the immunophillin group of proteins known for their property of binding to the immune-suppressant drug cyclosporine A. This particular protein is unusual due to the presence of an additional WD domain (along the traditional PPIase domain) experimentally proven to exist in *A. thaliana* (*AT3G44600 – CYP71/AtCYP71*) and *O. sativa* (*OS08G0557500/LOC_Os08g44330*) [33]. Due to its ability to modulate the distribution of FAS1 and LHP1 on chromatin in plants, loss of this gene function causes drastic pleiotropic phenotypic defects [34]. While less is known about cyclophylin A in wheat, some information is available about cyclophylin B [35]. The wheat gene (TRAES_7DS_8020BEEC2) in this clique had 16 original GO terms and was enriched by our model with 35 new GO terms with an average GO score of 1.12. The highest scored new GO term assigned to this gene was *protein peptidyl-prolyl isomerization* (GO:0000413).

**Clique 2** includes 10 genes potentially representing one (NMAT1) of the four nuclear maturase genes (NMAT1 to 4) encoding mitochondrial proteins in plants. In Arabidopsis, NMAT1 (AT1G30010) functions in the trans-splicing of *nad1* intron 1, and has a role in cis-splicing of *nad2* intron 1 and *nad4* intron 2 [36]. While no NMAT1 gene was experimentally confirmed in wheat yet, recent studies in wheat mitochondria showed that *nad2* intron 1, *nad1* intron 2 and *cox2* shifted from a predominantly hydrolytic pathway at room temperature to alternative pathways in the cold [37]. The wheat gene (TRAES_7DL_659883F3D) in this clique had 4 original GO terms and was enriched by our model with 5 new GO terms with an average GO score of 3.32. All 5 new GO terms were equally scored and represent *mitochondrion* (GO:0005739), *seed germination* (GO:0009845), *seedling development* (GO:0090351), *Group III intron splicing* (GO:0000374) and *vegetative to reproductive phase transition of meristem* (GO:0010228).

Genes in **Clique 3** show high DNA and protein sequence similarity with the *A. thaliana* “*chlororespiratory reduction 6*” (*AT2G47910 – CRR6*) chloroplast thylakoid membrane protein. In Arabidopsis, this protein is required for the assembly of the NAD(P)H dehydrogenase complex of the photosynthetic electron transport chain. A suite of recent studies in wheat revealed how photosystem 1 and 2 activity is influenced by various stress factors such as heat [38,39] and draught [40], nevertheless no genes were clearly identified as key factors in the corresponding pathways. The wheat gene (TRAES_6AS_87906149C) in this clique had 4 original GO terms and was enriched by our model with 2 new GO terms with an average GO score of 0.92. The two new GO terms are *iron-sulfur cluster assembly* (GO:0016226) and *aromatic amino acid family* (GO:0009073).

Genes in **Clique 4** are highly similar sequence-wise with *A. thaliana* “*ABC-2 type transporter family protein*” (AT3G13220) ATP-binding cassette transporter G26 (ABCG26) involved in tapetal cell and pollen development. This gene is required for male fertility and pollen exine formation. The predicted ortholog in rice (OS06G0607700) was identified in a 2013 study [41] as one of the important components for sporopollenin synthesis and secretion, functioning as transporter of sporopollenin precursors, translocating its substrates from tapetal cells to the developmental microspores. This gene plays a major role in the anther development and male sterility in rice. While studies of pollen formation at the phenotype level in *T. aestivum* were reported as early as 1986 [42], further work is needed to reveal the complex gene-based mechanisms that control the pollen development process in wheat. The wheat gene (TRAES_7DL_439CC6EA0) in this clique had 9 original GO terms and was enriched by our model with 1 new GO term with a GO score of 2.32. The new GO term turned out to be an obsolete one (ATP catabolic process - GO:0006200) currently replaced by ATPase activity (GO:0016887), which was already part of the original GO terms. Thus no real enrichment was obtained for this wheat gene.

**Clique 5** contains genes with a high degree of similarity with *A. thaliana* “*methyltransferases*” FIO1/FIONA1 (AT2G21070) gene, which is a genetic regulator of period length in the plant’s circadian clock. The gene is located in the nucleus and is involved in methyltransferase activity in flowering, circadian rhythm and photoperiodism. Other methyltransferase genes were isolated and characterized in monocots such as maize [43] and rice [44]. In wheat, five homologous cDNA sequences were connected with methyltransferase activity [45] and their expression patterns were studied. The *Cab-1* gene was also identified as a circadian clock regulator in wheat in 1988 [46]. The wheat gene (TRAES_6AS_AD173C5A3) in this clique had 1 original GO term and was enriched by our model with 4 new GO terms with an average GO score of 1.69. The new GO terms are *methylation* (GO:0032259), *nucleus* (GO:0005634), *circadian rhythm* (GO:0007623) and *photoperiodism, flowering* (GO:0048573).

Genes in **Clique 6** are related to *A. thaliana* “*Rhodanese/Cell cycle control phosphatase superfamily protein*” (AT2G40760) located in chloroplast and involved in aging. Genes with similar functionality were identified in other plants such as a predicted orthologous gene in maize (GRMZM2G087671), which was previously identified as a target gene of draught-responsive microRNAs [47]. In wheat, a previous study [48] reported the existence of a full-length cDNA sequence named TaTST (Triticum aestivum thiosulfate sulfurtransferase) with role in powdery mildew resistance mapped on the short arm of 6B chromosomes of wheat through Southern blot and GSP-PCR using Chinese Spring nullisomic/tetrasomic lines and ditelosomic lines. The wheat gene (TRAES_6AS_FD8F6B539) in this clique was not previously annotated and was enriched by our model with 1 new GO term with a GO score of 0.95. The new GO term is *aging* (GO:0007568).

**Clique 7** includes genes related to *A. thaliana* “*3-oxo-5-alpha-steroid 4-dehydrogenase family protein*” (AT3G55360 – CER10), which is located in the endoplasmic reticulum. This gene is an Enoyl-CoA reductase involved in all very long chain fatty acids (VLCFA) elongation reactions that are required for cuticular wax, storage lipid and sphingolipid metabolism. AT3G55360 apparently encodes the sole enoyl reductase activity associated with microsomal fatty acid elongation in Arabidopsis [49]. The Affymetrix wheat probe set Ta.28682.2.S1_x_at is associated with the CER10 Arabidopsis gene. This Arabidopsis gene shows a high level of similarity with the orthologous wheat gene TRAES_3B_90F2B79E9 in Clique 7, which was experimentally assigned the putative function “*Enoyl-CoA reductase*” in a 2010 study focused on changes in properties of wheat leaf cuticle during interactions with Hessian fly [50]. The wheat gene (TRAES_3B_90F2B79E9) in this clique had 4 original GO terms and was enriched by our model with 9 new GO terms with an average GO score of 1.54. The GO terms with the highest score (3.32) are *fatty acid elongase activity* (GO:0009922), *trans-2-enoyl-CoA reductase (NADPH) activity* (GO:0019166) and *plasma membrane* (GO:0005886).

Among the 13 cliques of size 10, **Clique 8** includes 1-to-1 orthologous genes with less specific annotation. The annotations corresponding to the *A. thaliana* gene AT3G55760 leads to an unknown protein located in the chloroplast stroma and expressed in 16 plant structures. The wheat gene (TRAES_4BS_2159A428F) in this clique had 2 original GO terms and was not enriched by our model.

**Clique 9** includes genes with high sequence similarity with *A. thaliana* “*Jumonji domain-containing protein 22*” (AT5G06550). This gene encodes a hairless (HR) demethylase that acts as a positive regulator of seed germination in the PHYB-PIL5-SOM pathway. GramineaeTFDB lists the wheat *tplb0016n18* transcript factor as one of the 4 members of the Jumonji family proteins. This TF is homologous with the wheat Ensembl gene model TRAES_7DL_96FFFB41E (reciprocal best BLAST *e-values* equal with 1e-157 and 6e-163 for *blastp* and 0 for *blastn*) that we identified as being part of Clique 9 and is reported to have orthologous sequences in 6 plant species (*A. thaliana*, *B. distachyon*, *H. vulgare*, *O. sativa*, *S. bicolor* and *Z. mays*) – all being 1-to-1 orthologs in Clique 9. The wheat gene (TRAES_7DL_96FFFB41E) in this clique had 5 original GO terms and was enriched by our model with 4 new GO terms with an average GO score of 1.08. The new GO terms are *regulation of flower development* (GO:0009909), *protein targeting to mitochondrion* (GO:0006626), *cell surface receptor signalling pathway* (GO:0007166) and *regulation of transcription, DNA-templated* (GO:0006355).

**Clique 10** consists of genes similar to a putative *A. thaliana* “*GMP synthase (glutamine-hydrolyzing)/glutamine amidotransferase*” (AT1G63660). Based on the limited available annotation, this gene plays a role in asparagine synthase (glutamine-hydrolyzing) activity, catalytic activity, GMP synthase (glutamine-hydrolyzing) activity and ATP binding. Two sets of genes in rice and Arabidopsis (housekeeping and tissue specific) which have evolved under contrasting evolutionary constraints include the rice gene OS08G0326600 as an ortholog of AT1G63660 [51]. The corresponding wheat Affymetrix probe set id (Ta.3136.1.S1_at) for the Ensembl wheat gene model TRAES_6AS_E6DEE586C is listed on the PlaNet [52] website as being co-expressed with the aforementioned rice gene. This suggests that TRAES_6AS_E6DEE586C is a good candidate for a wheat GMP synthase gene. The wheat gene (TRAES_6AS_E6DEE586C) in this clique had 7 original GO terms and was enriched by our model with 4 new GO terms with an average GO score of 1.54. The new GO terms are *cytosol* (GO:0005829), *RNA methylation* (GO:0001510), *protein import into nucleus* (GO:0006606) and *pyrimidine ribonucleotide biosynthetic process* (GO:0009220).

Genes in **Clique 11** are 1-to-1 orthologs with *A. thaliana* gene AT4G35870, characterized in TAIR as an “*early-responsive to dehydration stress protein (ERD4)*”, which, interestingly, coincides with the description of another Arabidopsis gene, namely AT1G30360 and in UniProt as a “*CSC1-like protein*”, which is located in the cell membrane and is involved in protein targeting to vacuole. The corresponding AT4G35870 protein was experimentally determined to be involved in vacuolar sorting of storage proteins (AtGFS10) [53]. The wheat gene (TRAES_4DL_11B05CF85) in this clique had 1 original GO term and was enriched by our model with 2 new GO terms with an average GO score of 2.33. The new GO terms are *protein targeting to vacuole* (GO:0006623) and *integral component of membrane* (GO:0016021).

**Clique 12** consists of highly similar genes with *A. thaliana* “*NAD(P)-binding Rossmann-fold superfamily protein*” (AT4G35250 - HCF244), which is located in chloroplast and manifests a binding and catalytic activity. Interestingly, the Arabidopsis gene was identified as an ortholog of the Ycf39 (CyanoBase designation Slr0399), which was originally identified in a screen for suppressor mutants that restored the ability of a D2 mutant of Synechocystis 6803 to bind the bound plastoquinone, QA, and was suggested to be involved in delivering plastoquinone to Photosystem II during assembly [54,55]. The wheat gene (TRAES_7DS_E3B38CA36) in this clique was not previously annotated and was enriched by our model with 9 new GO terms with an average GO score of 2.09. The 3 GO terms with the highest score are *chloroplast thylakoid* (GO:0009534), *translation initiation factor activity* (GO:0003743) and *photosystem II assembly* (GO:0010207).

Genes in **Clique 13** are highly similar with *A. thaliana* “*RAD3-like DNA-binding helicase protein*” (AT1G03190 - UVH6/AtXPD). This gene acts as a negative regulator for plant response to UV damage and heat, which trigger tissue death and reduced chloroplast function. The gene functions in DNA repair and it is essential for plant growth [56]. According to KEGG, the gene is involved in two pathways, namely the basal transcription factors pathway (*ath03022*) and the nucleotide excision repair pathway (*ath03420*). The wheat gene (TRAES_1AS_A25EED9EA) in this clique had 9 original GO terms and was enriched by our model with 9 new GO terms with an average GO score of 1.12. All new GO terms received the same scores and include *heat acclimation* (GO:0010286), *response to high light intensity* (GO:0009644), *transcription from RNA polymerase II promoter* (GO:0006366) and *RNA splicing, via endonucleolytic cleavage and ligation* (GO:0000394).

### Co-expression and expression profile similarity for genes in cliques of size 10

We verified the similarity of expression profiles for genes in cliques of size 10 using the Expressolog Tree Viewer [57] from the Bio-Analytic Resource (BAR) for Plant Biology available at University of Toronto [see Additional file 1: Table S3 ]. Expression datasets available in GEO for 4 plant species were used, such as: *A. thaliana* (AtGenExpress data series of Schmid et al., 2005 [58]), *O. sativa* (GEO accession numbers GSE7951 and GSE6893), *H. vulgare* (GEO accession number GSE16754) and *Z. mays* (PlexDB experiment number ZM37). Regrettably, wheat is not included yet in their database.

The Arabidopsis, rice and maize genes in all but one (clique 13) of the cliques of size 10 were identified as expressologs. The average expression profile similarity SCC scores (Spearman Correlation Coefficients) equal to 0.21 (std. dev. = 0.12) for Arabidopsis vs. rice and 0.37 (std. dev. = 0.24) for Arabidopsis vs. maize. For clique 13, only Arabidopsis and maize genes were identified as expressologs with SCC = 0.15. In 9 of the 13 cliques of size 10 the barley genes were also identified as being expressologs with the corresponding Arabidopsis, rice and maize genes. In the remaining 4 cliques, no barley expressologs were identified. Overall, the similar expression profile evidence for genes in 4 out of 10 plant species suggests that our cliques of 1-to-1 orthologs are well defined.

To facilitate the connexion between the previous analysis and wheat genes, we investigated the co-expression of genes in cliques of size 10 using the “*Standard analysis*” NetworkComparer approach in PlaNet [52]. PlaNet includes information from 7 plant species, of which 5 overlap with the ones used in our study: Arabidopsis, barley, brachypodium, rice and wheat. The “*Standard analysis*” NetworkComparer approach compares a gene of interest with other genes (represented by microarray probe sets) belonging to the PFAM family of the query. These probe sets are then used to generate an ancestral network, which depicts conserved co-expression relationships across selected probe sets and the identity of transcripts constituting conserved PFAMs are revealed. For 9 out of 13 cliques of size 10, wheat genes shared conserved Pfam domains with genes in up to 4 other plant species (Arabidopsis, barley, brachypodium and rice) from the PlaNet database [see Additional file 3]. In 4 of those cases, all 5 genes shared the same Pfam domains. Similar with our gene ontology analysis discussed above, no genes in Clique 8 were identified to share conserved Pfam domains. In addition, we used the Pfam2GO mappings of Reviewed Computational Analysis (RCA) annotations provided by the Gene Ontology Consortium to map 23 (22 original and 1 new) GO terms from a total of 147 original and new GO terms corresponding to the 13 wheat genes in cliques of size 10. The new GO term “*protein peptidyl-prolyl isomerization*” (GO:0000413) confirmed by this approach corresponded to the wheat gene *TRAES_7DS_8020BEEC2* in Clique 1, while the 22 original GO terms characterised wheat genes in cliques 1, 2, 4, 5, 7, 10, 11 and 13.

### Gene ontology characterization

**Figure 7:**
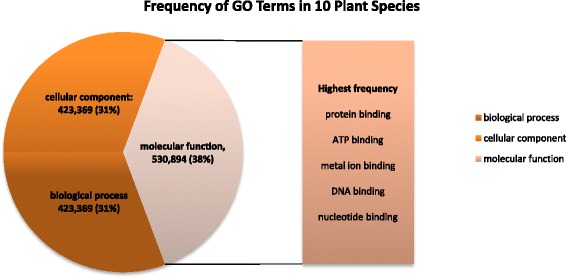
Distribution of GO terms in the 3 main categories for the 10 plant species considered in this work.

We provide in this section a generic view of the functional annotations available for the 10 plants species considered in this study. We use information extracted from Ensembl Plants release 22 and the Gene Ontology Consortium databases. The overall frequency of GO terms for the 10 plant species considered in this work is depicted in Figure 7. The GO terms with the highest occurrence in each of the three main sub-ontologies (biological process, cellular component, and molecular function) are highlighted in Additional file 2: Figures S3-S5.

Overall, for the 10 plant species, the top five high frequency GO terms in the “*molecular function*” group are: *protein binding* (GO:0005515), *ATP binding* (GO:0005524), *metal ion binding* (GO:0046872), *DNA binding* (GO:0003677) and *nucleotide binding* (GO:0000166). The top five in “*cellular component*” GO terms are: *membrane* (GO:0016020), *nucleus* (GO:0005634), *integral to membrane* (GO:0016021), *plasma membrane* (GO:0005886) and *chloroplast* (GO:0009507). Similarly, the five highly occurring “*biological process*” GO terms for the 10 plant species are: *oxidation-reduction process* (GO:0055114), *protein phosphorylation* (GO:0006468), *metabolic process* (GO:0008152), *regulation of transcription - DNA-dependent* (GO:0006355) and *biological process* (GO:0008150).

For cliques of size 10, “cellular component” GO terms [see Additional file 2: Figure S6] such as: *nucleus* (GO:0005634, 34 times), *chloroplast* (GO:0009507, 29 times), *membrane* (GO:0016020, 28 times) and *chloroplast stroma* (GO:0009570, 20 times) occur more frequently among the participant genes. “Molecular function” GO terms [see Additional file 2: Figure S7] have also significantly high occurrence, such as *ATP binding* (GO:0005524, 30 times), *protein binding* (GO:0005515, 21 times) and *nucleotide binding* (GO:0000166, 19 times), while “biological process” GO terms [see Additional file 2: Figure S8] have medium to lower occurrences, the most prevalent being *regulation of flower development* (GO:0009909, 14 times), *protein folding* (GO:0006457, 14 times), *vegetative to reproductive phase transition of meristem* (GO:0010228, 13 times) and *seed germination* (GO:0009845, 13 times).

### Gene Ontology (GO) enrichment model

Based on the gene ontology acquired from Ensembl Plants we propose the following procedure for enriching the annotation of wheat genes using information extrapolated from 1-to-1 orthologs with 9 other plant species.

As first step, we assign to each gene in all 10 plant species the corresponding GO terms found in Ensembl Plants. Table 3 provides information related to the number of genes in each species that has associated GO terms. Among all species *A. thaliana* has the highest percentage of annotated genes (93%), while *O. sativa*, *T. aestivum* and *T. urartu* have the lowest percentages (57%, 61% and 58%, respectively). For *T. aestivum*, 60,577 out of 98,779 putative genes are annotated, leaving 39% without any annotation. For cliques of size ten, 11 out of 13 wheat genes have existing annotation (associated GO terms) in Ensembl Plants release 22. The two un-annotated wheat genes are TRAES_6AS_FD8F6B539 in Clique 6 associated with the “*Rhodanese/Cell cycle control phosphatase superfamily protein*” and TRAES_7DS_E3B38CA36, respectively, in Clique 12 associated with the “*NAD(P)-binding Rossmann-fold superfamily protein*” in *A. thaliana*.

**Table 3:** **Status of currently known annotated genes (proteins) in 10 plant species**

**Species**	**Total num. genes**	**Num. annotated genes**	**Num. unannotated genes (%)**
*Aegilops tauschii*	33,849	21,552	12,297 (36%)
*Arabidopsis thaliana*	27,416	25,431	1,985 (7%)
*Brachypodium distachyon*	26,552	18,618	7,934 (30%)
*Brassica rapa*	41,018	28,015	13,003 (32%)
*Hordeum vulgare*	24,211	16,858	7,353 (30%)
*Oryza sativa*	35,679	20,492	15,187 (43%)
*Sorghum bicolor*	34,496	22,797	11,699 (34%)
*Triticum aestivum*	98,779	60,577	38,202 (39%)
*Triticum urartu*	33,424	19,473	13,951 (42%)
*Zea mays*	38,741	25,552	13,189 (34%)

Data was extracted from Ensembl Plants release 22.

The second step consists of calculating the gene ontology scores (Equation 2) based on the phylogenetic proximity between genes in two species, the overall hierarchy of gene ontology and the predicted orthology relationships. For each wheat gene which belongs to a clique of size at least 3, we assign a new GO term if the GO score is above the 0.5 threshold. The genes in cliques of size at least 3 have a total of 107,997 GO terms associations. This step selects 80,067 new GO term assignments with scores equal to or higher than 0.5, leading to an overall cumulative improvement of 47% compared to the existing gene-GO term associations (171,488).

For cliques of size ten, 12 out of 13 wheat genes acquired a total of 150 new GO term associations (cumulative improvement of 115%) over the existing 130 GO terms.

The third step consists of a refinement for assignment of GO terms by keeping only those new terms that add more information (higher specificity) to the gene, i.e. we accept only those new GO terms that reside deeper (closer to a leaf node in the graph) or on a different path than the original annotations in the overall GO graph hierarchy. This will also ensure that no “place holder” root GO terms (molecular function, cellular component and biological process) will be selected for those genes where annotations already exist along the corresponding paths in the GO graph. Nevertheless, root GO terms will be accepted when no annotation is available for a given gene or if the available annotation follows a different path corresponding to a different root GO term in the GO term graph. Based on this approach, out of 80,067 new GO term assignments with scores equal to or higher than 0.5 assigned at step 2, only 25,607 new GO terms add more information to the existing annotations (only 152 root GO terms). For these, the GO term path length varies between 2 and 15 with a mean around 6.4 [see Additional file 2: Figures S9 and S10] and the GO scores span the [0.5, 3.3] interval with the mean around 1.1 [see Additional file 2: Figures S11 and S12]. This process leads to an overall GO term assignment increase of 15% compared to the original annotation (171,488 GO terms). A total of 7,838 wheat genes were annotated with newly assigned GO terms, leading to an overall increase of 8% with respect to the total number of wheat genes in Ensembl Plants release 22. Out of those 7,838 genes, 2,139 had no previous annotation [see Additional file 4] of which only 145 represent root GO terms (16 biological process - GO:0008150 and 129 molecular function - GO:0003674).

For cliques of size 10, only 85 out of 150 new GO terms are more specific and enrich the annotations based on their GO graph paths. This leads to an overall improvement of 65% compared to existing annotations (130 GO terms). Two wheat genes, namely *TRAES_6AS_FD8F6B539* and *TRAES_7DS_E3B38CA36* [see Additional file 5], out of 13 had no previous known annotations and were enriched with 1 (GO:0007568 - *aging*) and 9 (GO:0003743 - *translation initiation factor activity*, GO:0009507 - *chloroplast*, GO:0009534 - *chloroplast thylakoid*, GO:0016117 - *carotenoid biosynthetic process*, GO:0019288 - *isopentenyl diphosphate biosynthetic process, methylerythritol 4-phosphate pathway*, GO:0019684 - *photosynthesis, light reaction*, GO:0006364 - *rRNA processing*, GO:0010114 - *response to red light*, and GO:0010207 - *photosystem II assembly*) new GO terms, respectively.

**Figure 8:**
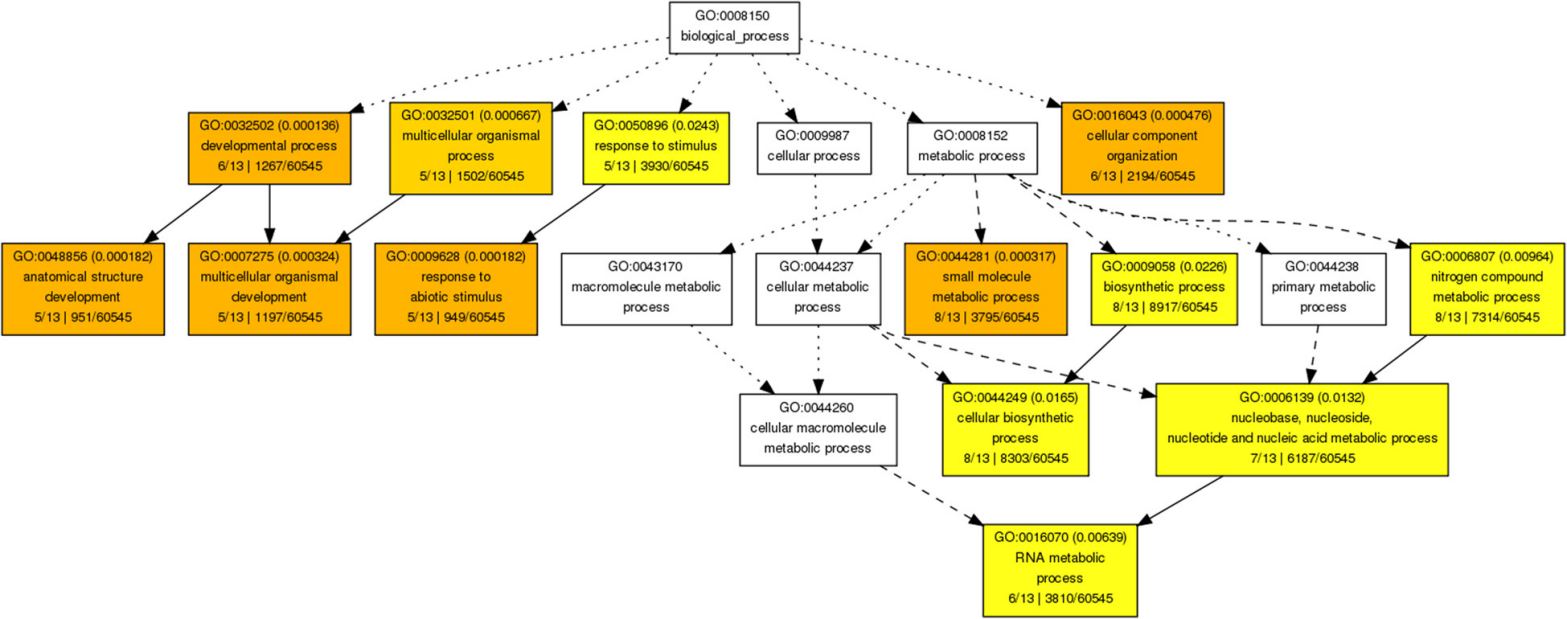
AgriGO display of statistically significant Biological Process GO terms for the cliques of size 10. Inside the box of the significant GO terms, the information includes: GO term, adjusted p-value, GO description, item number mapping the GO in the query list and background, and total number of query list and background.

**Figure 9:**
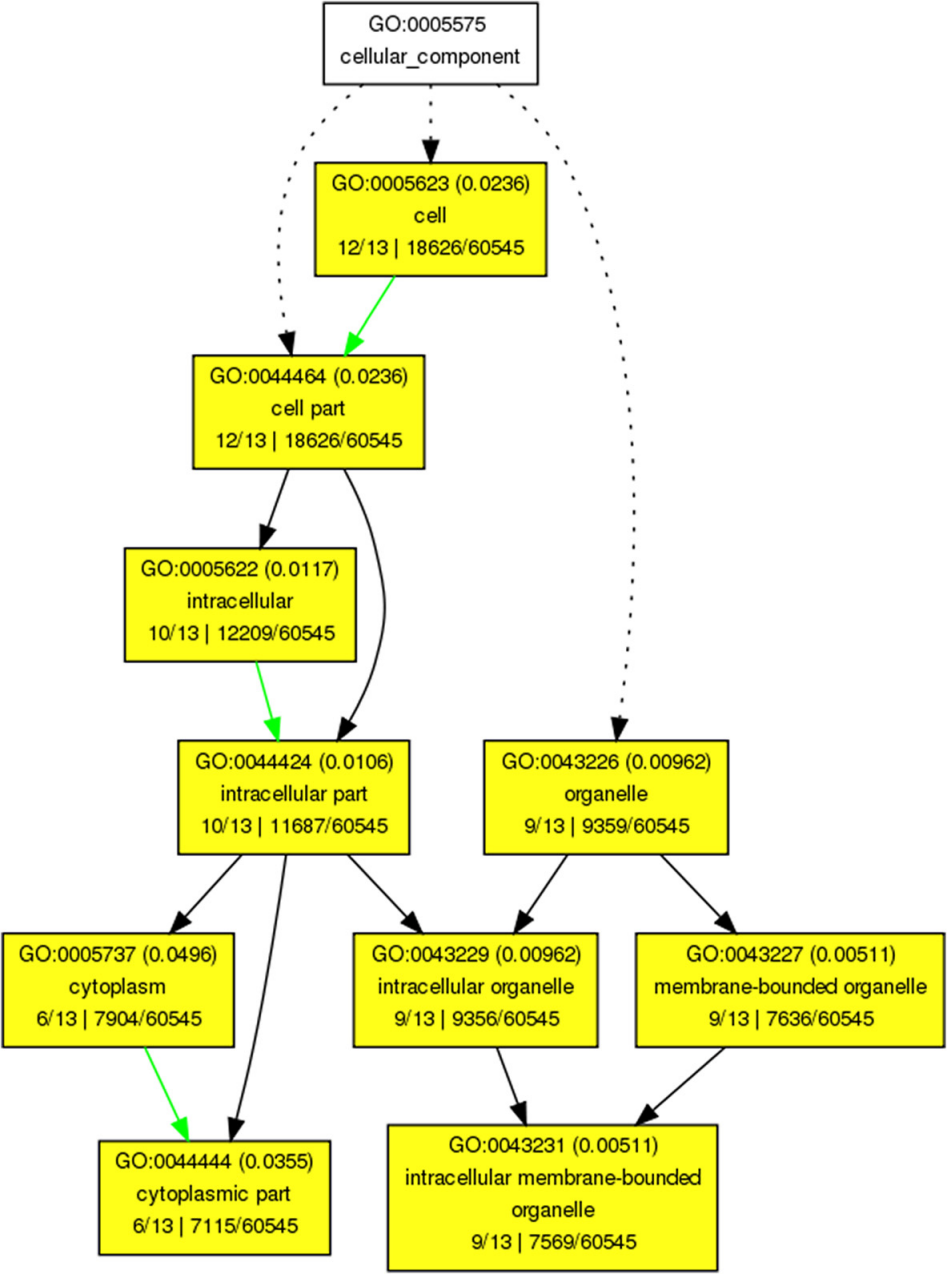
AgriGO display of statistically significant Cellular Component GO terms for the cliques of size 10. Inside the box of the significant GO terms, the information includes: GO term, adjusted p-value, GO description, item number mapping the GO in the query list and background, and total number of query list and background.

**Figure 10:**
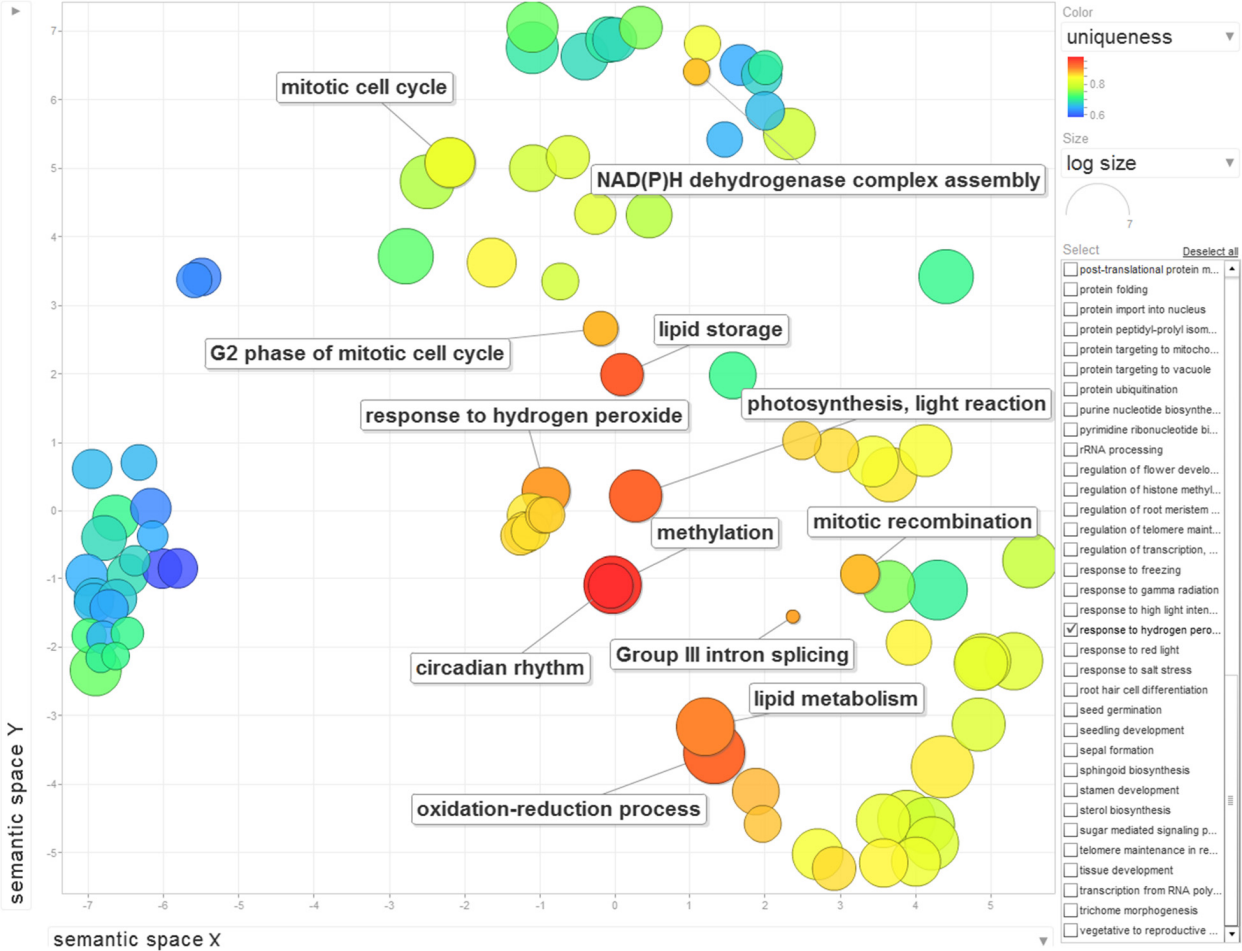
ReviGO display of 96 Biological Process GO terms for the cliques of size 10. The GO terms with higher uniquenes are displayed in shades of red while the ones with lower uniqueness are displayed with shades of blue. The GO terms with higher semantic similarity are closer on the plot.

**Figure 11:**
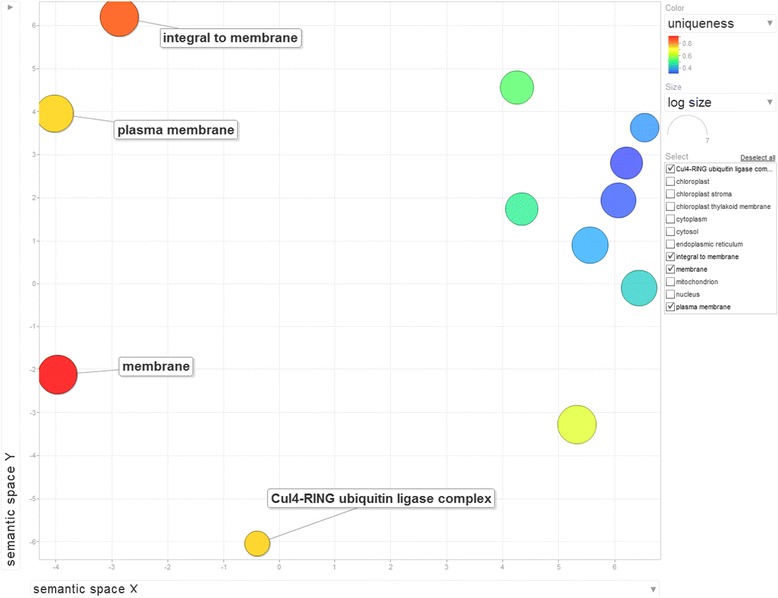
ReviGO display of 22 Cellular Component GO terms for the cliques of size 10.

**Figure 12:**
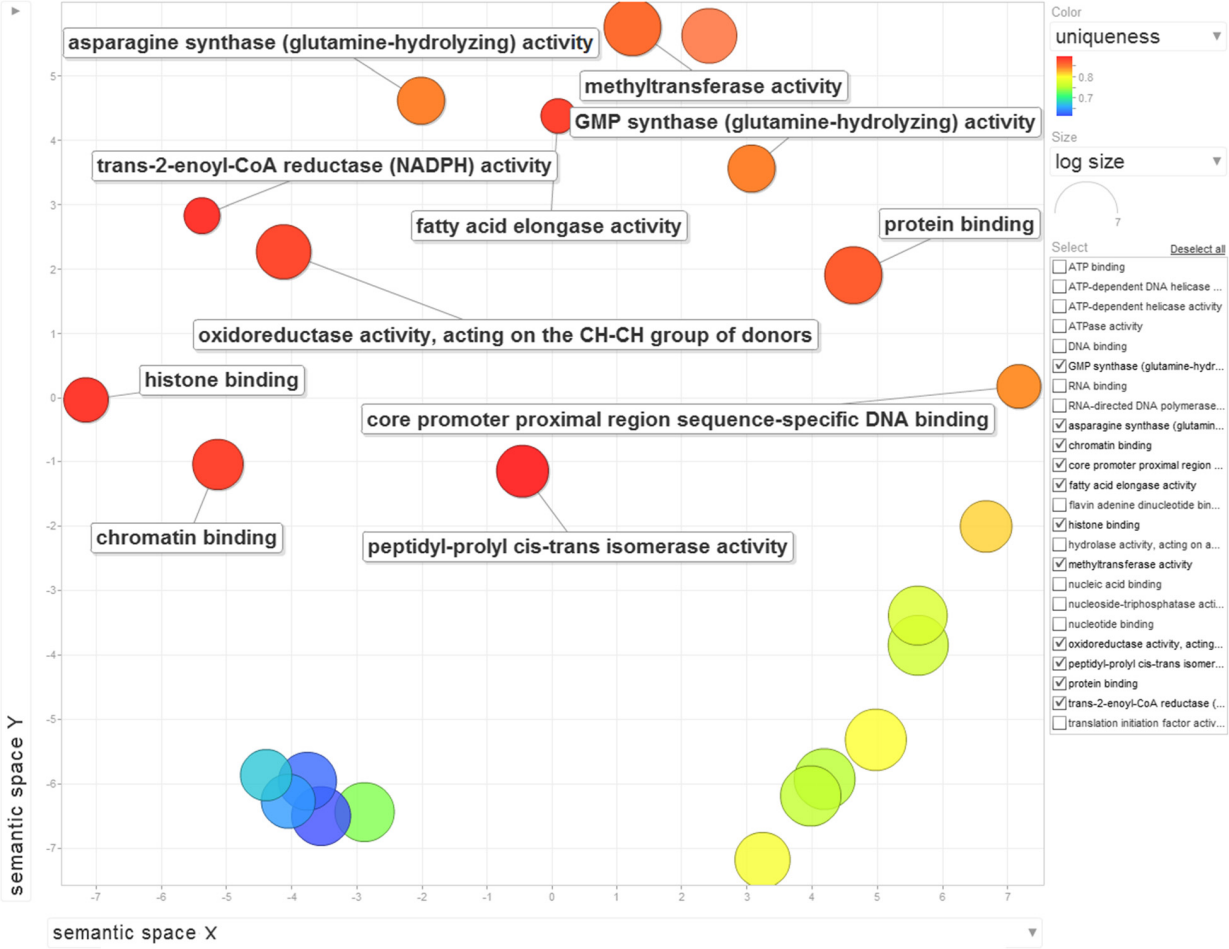
ReviGO display of 29 Molecular Function GO terms for the cliques of size 10.

While orthology relationships have been identified between genes in pairwise species, no functional relationship is expected to be found among sets of genes belonging to cliques of the same size. In other words, clique size does not determine functional similarities among genes. Thus we can only provide a descriptive analysis of the gene ontologies associated with those genes. We perform Singular Enrichment Analysis (SEA) [59] in the absence of the traditional gene expression information (thus no *p-value* calculations apply in this case) using as input the GO terms for the 13 wheat genes in cliques of size 10 and the whole Ensembl Plants wheat GO set as reference. Using AgriGO [28] analysis, a set of 23 enriched GO terms were identified (Figures 8 and 9) for the genes in all 13 cliques of size 10. The ReviGO [60] analysis also performed in the absence of additional gene expression information identifies semantically similar GO terms (using UniProt as reference database) displayed in a 2D scatter plot based on an eigenvalue decomposition of the terms' pairwise distance matrix followed by a stress minimization step, which iteratively improves the agreement between the GO terms' semantic similarities and their closeness (Figures 10, 11 and 12). For cliques of size 10, five pairs of GO terms (4 biological process, 4 molecular function and 2 cellular component GO terms) were identified as having similar and thus redundant functions and were clustered together. For instance, the biological process GO terms characteristic to wheat gene TRAES_7DS_8020BEEC2 in clique 1, namely “*sepal formation*” (GO:0048453) and “*petal formation*” (GO:0048451), are similar (*SimRel* = 0.65 for each) and thus they are merged in a cluster represented by the first term. Interestingly, the “*petal formation*” GO term was newly assigned to this wheat gene by our enrichment model. Similarly, for the same wheat gene, “*carpel development*” (GO:0048440) and “*stamen development*” (GO:0048443) were merged in a cluster represented by the former GO term, both terms being already part of the gene ontology information available in Ensembl Plants for this wheat gene.

## Conclusions

We propose a novel annotation model for wheat genes based on 1-to-1 cliques of orthologs, existing gene ontologies from 9 other plant species and their phylogenetic relationship. Our annotation model relies on the intersection of 1-to-1 orthologs predictions based on DNA and protein sequences encoded by the same gene. Large cliques of orthologs combined with an additive scoring scheme based on phylogenetic distances between plant species provide the mechanism for gene ontology knowledge transfer from orthologous genes in other well annotated plant species to wheat genes.

In addition, we provided experimental validation and analysis of genes with similar expression profiles in Arabidopsis, barley, maize and rice and evidence that our cliques of orthologs are valid. Our model demonstrated that wheat gene functional annotations can be enriched via cliques of 1-to-1 orthologs, gene ontology information and phylogenetic relationships among considered species.

To further bridge the gap between newly sequenced and completely characterized wheat genes and proteins, a large number of validated annotations is required from the experimental community. These extremely valuable manual annotations can be in turn integrated into automatic computational annotation pipelines and models such as the one presented here to further increase the quality, throughput and understanding of the deluge of information generated today.

## Additional files

Additional file 1:
**Additional tables.** Additional file 1 includes 3 additional tables that provide additional support for our findings. Additional file 2:
**Additional figures.** Additional file 2 includes 12 additional figures that provide additional support for our findings. Additional file 3:
**PlaNet Analysis - Pfam domains shared by genes from 5 plant species.** The Excel file contains 3 worksheets. First worksheet presents PFam domains shared by genes in 5 plant species (*A. thaliana*, *B. distachyon*, *H. vulgare*, *O. sativa* and *T. aestivum*) identified using PlaNet. The second worksheet includes GO terms associated to each PFam domain associated with the 13 wheat genes in the cliques of size 10. The third worksheet contains a list of SupFam supra-domains and corresponding GO terms for wheat genes in each clique of size 10 (highlighted with alternate colours). For each GO term, we provide the GO name, GO sub-ontologies, information content, annotation origin and the associated label (new, original or **n**ot **f**ound = **n.f.**). Additional file 4:
**New GO term annotation for 2,139 previously un-annotated wheat genes.** The Excel file contains one worksheet with 2 columns. First column represents the wheat Ensembl Gene ID without previous annotation and the second column contains the newly assigned GO terms. Additional file 5:
**GO term assignments for genes in the cliques of size 10.** The Excel file contains 2 tables in separate worksheets. First table includes existing and new GO terms associated with wheat genes in 13 cliques of size 10. Second table provides detailed information such as GO graph depth, GO score, GO graph path and description for each GO term associated with the 13 wheat genes in cliques of size 10.
