# Supplementary material for: Enrichment of Triticum aestivum gene annotations using ortholog cliques and gene ontologies in other plants
Source: BMC Genomics. 2015 Apr 15;16(1):299. doi: 10.1186/s12864-015-1496-2 (PMC4426649; doi:10.1186/s12864-015-1496-2)
Supplement: Additional file 1: — Additional tables. Additional file 1 includes 3 additional tables that provide additional support for our findings. [file 12864_2015_1496_MOESM1_ESM.docx]

Additional file 1 – Additional tables

Table 1. Number of pairwise species RBBHs predicted using coding DNA sequences (top values), proteins (middle values) and their intersection (bold face, bottom values). ATA = *Aegilops tauschii*, ATH = *Arabidopsis thaliana*, BDI = *Brachypodium distachion*, BRA = *Brassica rapa*, HVU = *Hordeum vulgare*, OSA = *Oryza sativa*, SBI = *Sorghum bicolor*, TAE = *Triticum aestivum*, TUR = *Triticum urartu*, ZMA = *Zea mays*.

|  | **ATH** | **BDI** | **BRA** | **HVU** | **OSA** | **SBI** | **TAE** | **TUR** | **ZMA** |
| --- | --- | --- | --- | --- | --- | --- | --- | --- | --- |
| **ATA** | 5,207  8,978  **2754** | 16,534  14,877  **13,066** | 5,960  8,948  **5,960** | 16,203  13,883  **12,551** | 15,591  13,267  **10,757** | 15,433  13,746  **11,433** | 21,814  18,737  **14,347** | 19,816  17,614  **16,079** | 14,182  12,618  **9,550** |
| **ATH** | - | 6,035  10,740  **3,581** | 19,590  18,945  **15,345** | 5,095  9,249  **2,819** | 5,978  10,273  **3,184** | 6,086  10,721  **3,518** | 6,422  10,474  **1,530** | 5,178  8,943  **2,762** | 5,739  10,307  **2,840** |
| **BDI** | - | - | 6,805  10,606  **3,039** | 16,472  14,683  **13,261** | 17,968  16,318  **14,235** | 17,903  17,133  **15,175** | 18,572  17,176  **11,125** | 16,143  14,550  **12,745** | 16,465  15,522  **12,580** |
| **BRA** | - | - | - | 5,815  9,116  **2,328** | 6,846  10,140  **2,725** | 6,860  10,565  **2,981** | 7,506  10,401  **1,347** | 5,850  8,874  **2,330** | 6,632  10,172  **2,457** |
| **HVU** | - | - | - | - | 15,683  13,310  **10,988** | 15,625  13,885  **11,814** | 18,682  16,356  **10,462** | 15,955  13,668  **12,217** | 14,592  12,960  **9,960** |
| **OSA** | - | - | - | - | - | 18,693  16,688  **14,384** | 17,777  15,556  **8,790** | 15,256  13,298  **15,256** | 17,120  15,299  **11,945** |
| **SBI** | - | - | - | - | - | - | 17,715  16,237  **9,193** | 15,164  13,549  **11,156** | 19,713  18,238  **16,055** |
| **TAE** | - | - | - | - | - | - | - | 21,001  18,211  **13,069** | 16,516  15,041  **7,721** |
| **TUR** | - | - | - | - | - | - | - | - | 13,982  12,477  **9,340** |

Table 2. Functional annotation of 1-to-1 orthologous genes in the cliques of size 10.

| **Clique** | **Gene IDs with supporting annotation** | **Ensembl Plants release 22 - *A. thaliana* annotations** |
| --- | --- | --- |
| 1 | F775_06552  AT3G44600  BRADI3G42750  BRA019430  MLOC_38535  OS08G0557500  SB07G024330  TRIUR3_22015  GRMZM2G049525 | cyclophilin71 [Source: TAIR_LOCUS; Acc:AT3G44600] |
| 2 | F775_11186  AT1G30010  BRADI1G39140  BRA032352  MLOC_55696  SB01G044780  TRIUR3_27785 | Intron maturase, type II family protein [Source: TAIR_LOCUS; Acc:AT1G30010] |
| 3 | AT2G47910  BRA021447  MLOC_8265  OS08G0167500 | chlororespiratory reduction 6 [Source: TAIR_LOCUS; Acc:AT2G47910] |
| 4 | F775_06961  AT3G13220  BRADI1G36410  BRA039378  MLOC_66857  OS06G0607700 / LOC_Os06g40550  SB10G023750  GRMZM2G076526 | ABC-2 type transporter family protein [Source: TAIR_LOCUS; Acc:AT3G13220] |
| 5 | F775_11259  AT2G21070  BRA030323  OS02G0121200  SB04G001730  TRIUR3_05931 | FIO1; methyltransferases [Source: TAIR_LOCUS; Acc:AT2G21070] |
| 6 | AT2G40760  BRA016973  OS05G0323100 | Rhodanese/Cell cycle control phosphatase superfamily protein [Source: TAIR_LOCUS; Acc:AT2G40760] |
| 7 | F775_31011  AT3G55360  BRADI2G03297  BRA007154  MLOC_59964  OS01G0150000  TRIUR3_25686  GRMZM2G481843 | 3-oxo-5-alpha-steroid 4-dehydrogenase family protein [Source: TAIR_LOCUS; Acc:AT3G55360] |
| 8 | AT3G55760 | unknown protein; LOCATED IN: chloroplast stroma, chloroplast; EXPRESSED IN: 16 plant structures; EXPRESSED DURING: 10 growth stages; BEST Arabidopsis thaliana protein match is: unknown protein (TAIR:AT1G42430.2); Has 176 Blast hits to 125 proteins i /.../species: Archae - 0; Bacteria - 3; Metazoa - 19; Fungi - 9; Plants - 81; Viruses - 0; Other Eukaryotes - 64 (source: NCBI BLink). [Source: TAIR_LOCUS; Acc:AT3G55760] |
| 9 | F775_13173  AT5G06550  BRADI4G16020  BRA009205  MLOC_65909  OS11G0572800  SB05G022250  GRMZM2G078198 | CONTAINS InterPro DOMAIN/s: Transcription factor jumonji/aspartyl beta-hydroxylase (InterPro:IPR003347), F-box domain, Skp2-like (InterPro:IPR022364), Transcription factor jumonji (InterPro:IPR013129); BEST Arabidopsis thaliana protein match is: tra /.../ses, transferring glycosyl groups (TAIR:AT1G78280.1); Has 1762 Blast hits to 1747 proteins in 292 species: Archae - 0; Bacteria - 297; Metazoa - 877; Fungi - 168; Plants - 221; Viruses - 0; Other Eukaryotes - 199 (source: NCBI BLink). [Source: TAIR_LOCUS; Acc:AT5G06550] |
| 10 | F775_13768  AT1G63660  BRADI3G20590  BRA027795  OS08G0326600 | GMP synthase (glutamine-hydrolyzing), putative / glutamine amidotransferase, putative [Source: TAIR_LOCUS; Acc:AT1G63660] |
| 11 | F775_08440  AT4G35870  BRADI1G75920  BRA010507  TRIUR3_30140  GRMZM2G059891 | early-responsive to dehydration stress protein (ERD4) [Source: TAIR_LOCUS; Acc:AT4G35870] |
| 12 | F775_30997  AT4G35250  BRADI3G42580  BRA020809  MLOC_3618  OS08G0553800  TRIUR3_18085 | NAD(P)-binding Rossmann-fold superfamily protein [Source: TAIR_LOCUS; Acc:AT4G35250] |
| 13 | F775_08503  AT1G03190  BRADI2G36360  BRA030524  MLOC_66388 OS05G0144800  SB09G003450  TRIUR3_25030  GRMZM2G097605 | RAD3-like DNA-binding helicase protein [Source: TAIR_LOCUS; Acc:AT1G03190] |

Table 3: Validation for genes in the cliques of size 10 via BAR expressologs.

| **Clique** | **Genes** | **Ensembl Plants release 22 - *A. thaliana* annotations** | **Gene expression validation (BAR Expressolog – Arabidopsis developmental dataset)**  [**http://bar.utoronto.ca/expressolog_treeviewer/cgi-bin/expressolog_treeviewer.cgi**](http://bar.utoronto.ca/expressolog_treeviewer/cgi-bin/expressolog_treeviewer.cgi) | **Gene expression validation (BAR Expressolog – Arabidopsis stress dataset)**  [**http://bar.utoronto.ca/expressolog_treeviewer/cgi-bin/expressolog_treeviewer.cgi**](http://bar.utoronto.ca/expressolog_treeviewer/cgi-bin/expressolog_treeviewer.cgi) |
| --- | --- | --- | --- | --- |
| 1 | F775_06552  **AT3G44600**  BRADI3G42750  BRA019430  **MLOC_38535 / Contig16607_at**  **OS08G0557500 / LOC_OS08G44330**  SB07G024330  TRAES_7DS_8020BEEC2  TRIUR3_22015  **GRMZM2G049525** | cyclophilin71 [Source: TAIR_LOCUS; Acc:AT3G44600] | AT3G44600  OS08G0557500 / LOC_OS08G44330  MLOC_38535 / Contig16607_at  GRMZM2G049525 | AT3G44600  OS08G0557500 / LOC_OS08G44330 |
| 2 | F775_11186  **AT1G30010**  BRADI1G39140  BRA032352  MLOC_55696  **OS12G0407300 / LOC_OS12G21870**  SB01G044780  TRAES_7DL_659883F3D  TRIUR3_27785  **GRMZM2G023983** | Intron maturase, type II family protein [Source: TAIR_LOCUS; Acc:AT1G30010] | AT1G30010  Os12g0407300 / LOC_OS12G21870  GRMZM2G023983 | AT1G30010  Os12g0407300 / LOC_OS12G21870 |
| 3 | F775_08951  **AT2G47910**  BRADI3G16010  BRA021447  **MLOC_8265 / Contig12132_s_at**  **OS08G0167500 / LOC_OS08G07060**  SB02G024420  TRAES_6AS_87906149C  TRIUR3_09163  **GRMZM2G106164** | chlororespiratory reduction 6 [Source: TAIR_LOCUS; Acc:AT2G47910] | AT2G47910  OS08G0167500 / LOC_OS08G07060  MLOC_8265 / Contig12132_s_at  GRMZM2G106164 | AT2G47910  OS08G0167500 / LOC_OS08G07060 |
| 4 | F775_06961  **AT3G13220**  BRADI1G36410  BRA039378  MLOC_66857  **OS06G0607700 / LOC_OS06G40550**  SB10G023750  TRAES_7DL_439CC6EA0  TRIUR3_24106  **GRMZM2G076526** | ABC-2 type transporter family protein [Source: TAIR_LOCUS; Acc:AT3G13220] | AT3G13220  OS06G0607700 / LOC_OS06G40550  GRMZM2G076526 | AT3G13220  OS06G0607700 / LOC_OS06G40550 |
| 5 | F775_11259  **AT2G21070**  BRADI3G01970  BRA030323  **MLOC_14151 / Contig17346_at**  **OS02G0121200 / LOC_OS02G02880**  SB04G001730  TRAES_6AS_AD173C5A3  TRIUR3_05931  **GRMZM2G090156** | FIO1; methyltransferases [Source: TAIR_LOCUS; Acc:AT2G21070] | AT2G21070  OS02G0121200 / LOC_OS02G02880  MLOC_14151 / Contig17346_at  GRMZM2G090156 | AT2G21070  OS02G0121200 / LOC_OS02G02880 |
| 6 | F775_11739  **AT2G40760**  BRADI2G44260  BRA016973  **MLOC_63819 / Contig17763_at**  **OS05G0323100 / LOC_OS05G25780**  SB09G011890  TRAES_6AS_FD8F6B539  TRIUR3_17179  **GRMZM2G087671** | Rhodanese/Cell cycle control phosphatase superfamily protein [Source: TAIR_LOCUS; Acc:AT2G40760] | AT2G40760  OS05G0323100 / LOC_OS05G25780  n.a. / Contig17762_at  MLOC_63819 / Contig17763_at  GRMZM2G087671 | AT2G40760  OS05G0323100 / LOC_OS05G25780 |
| 7 | F775_31011  **AT3G55360**  BRADI2G03297  BRA007154  **MLOC_59964 / Contig4618_at**  **OS01G0150000 / LOC_OS01G05670**  SB03G006070  TRAES_3B_90F2B79E9  TRIUR3_25686  **GRMZM2G481843** | 3-oxo-5-alpha-steroid 4-dehydrogenase family protein [Source: TAIR_LOCUS; Acc:AT3G55360] | AT3G55360  OS01G0150000 / LOC_OS01G05670  MLOC_59964 / Contig4618_at  GRMZM2G481843 | AT3G55360  OS01G0150000 / LOC_OS01G05670 |
| 8 | F775_27767  **AT3G55760**  BRADI4G15010  BRA023783  **MLOC_9792** **/ Contig10009_at**  **OS11G0586300 / LOC_OS11G37560**  SB05G022830  TRAES_4BS_2159A428F  TRIUR3_31794  **GRMZM2G069092** | unknown protein; LOCATED IN: chloroplast stroma, chloroplast; EXPRESSED IN: 16 plant structures; EXPRESSED DURING: 10 growth stages; BEST Arabidopsis thaliana protein match is: unknown protein (TAIR:AT1G42430.2); Has 176 Blast hits to 125 proteins i /.../pecies: Archae - 0; Bacteria - 3; Metazoa - 19; Fungi - 9; Plants - 81; Viruses - 0; Other Eukaryotes - 64 (source: NCBI BLink). [Source: TAIR_LOCUS; Acc:AT3G55760] | AT3G55760  Os11g0586300 / LOC_OS11G37560  MLOC_9792 / Contig10009_at  GRMZM2G069092 | AT3G55760  Os11g0586300 / LOC_OS11G37560 |
| 9 | F775_13173  **AT5G06550**  BRADI4G16020  BRA009205  **MLOC_65909 / Contig16420_at**  **OS11G0572800 / LOC_OS11G36450**  SB05G022250  TRAES_7DL_96FFFB41E  TRIUR3_19243  **GRMZM2G078198** | CONTAINS InterPro DOMAIN/s: Transcription factor jumonji/aspartyl beta-hydroxylase (InterPro:IPR003347), F-box domain, Skp2-like (InterPro:IPR022364), Transcription factor jumonji (InterPro:IPR013129); BEST Arabidopsis thaliana protein match is: tra /.../ses, transferring glycosyl groups (TAIR:AT1G78280.1); Has 1762 Blast hits to 1747 proteins in 292 species: Archae - 0; Bacteria - 297; Metazoa - 877; Fungi - 168; Plants - 221; Viruses - 0; Other Eukaryotes - 199 (source: NCBI BLink). [Source: TAIR_LOCUS; Acc:AT5G06550] | AT5G06550  MLOC_65909 / Contig16420_at  OS11G0572800 / LOC_OS11G36450  GRMZM2G078198 | AT5G06550  OS11G0572800 / LOC_OS11G36450 |
| 10 | F775_13768  **AT1G63660**  BRADI3G20590  BRA027795  **MLOC_34318** **/ Contig8885_s_at**  **OS08G0326600 / LOC_OS08G23730**  SB06G033930  TRAES_6AS_E6DEE586C  TRIUR3_04203  **GRMZM2G136283** | GMP synthase (glutamine-hydrolyzing), putative / glutamine amidotransferase, putative [Source: TAIR_LOCUS; Acc:AT1G63660] | AT1G63660  OS08G0326600 / LOC_OS08G23730  MLOC_34318 / Contig8885_s_at  CONTIG8886 (Barley probe set id)  GRMZM2G136283 | AT1G63660  OS08G0326600 / LOC_OS08G23730 |
| 11 | F775_08440  **AT4G35870**  BRADI1G75920  BRA010507  MLOC_68300  **OS03G0137400 / LOC_OS03G04450**  SB01G047810  TRAES_4DL_11B05CF85  TRIUR3_30140  **GRMZM2G059891** | early-responsive to dehydration stress protein (ERD4) [Source: TAIR_LOCUS; Acc:AT4G35870] | AT4G35870  Os03g0137400 / LOC_OS03G04450  GRMZM2G059891 | AT4G35870  Os03g0137400 / LOC_OS03G04450 |
| 12 | F775_30997  **AT4G35250**  BRADI3G42580  BRA020809  **MLOC_3618** **/ Contig12596_at**  **OS08G0553800 / LOC_OS08G44000**  SB07G024590  TRAES_7DS_E3B38CA36  TRIUR3_18085  **GRMZM2G143917** | NAD(P)-binding Rossmann-fold superfamily protein [Source: TAIR_LOCUS; Acc:AT4G35250] | AT4G35250  Os08g0553800 / LOC_OS08G44000  MLOC_3618 / Contig12596_at  GRMZM2G143917 | AT4G35250  Os08g0553800 / LOC_OS08G44000 |
| 13 | F775_08503  **AT1G03190**  BRADI2G36360  BRA030524  MLOC_66388  **OS05G0144800 / LOC_OS05G05260**  SB09G003450  TRAES_1AS_A25EED9EA  TRIUR3_25030  **GRMZM2G097605** | RAD3-like DNA-binding helicase protein [Source: TAIR_LOCUS; Acc:AT1G03190] | AT1G03190  Os05g0144800 / LOC_OS05G05260  GRMZM2G097605 | AT1G03190  Os05g0144800 / LOC_OS05G05260 |
