# Supplementary material for: Enrichment of Triticum aestivum gene annotations using ortholog cliques and gene ontologies in other plants
Source: BMC Genomics. 2015 Apr 15;16(1):299. doi: 10.1186/s12864-015-1496-2 (PMC4426649; doi:10.1186/s12864-015-1496-2)
Supplement: Additional file 2: — Additional figures. Additional file 2 includes 12 additional figures that provide additional support for our findings. [file 12864_2015_1496_MOESM2_ESM.docx]

Additional file 2 – Additional figures

Figure 1. Number of 1-to-1 orthologs in mutually exclusive cliques with sizes between 3 and 10 for each plant species.

Figure 2. Histogram representing the number of orthologs in all 10 plant species included in the cliques with OCLs from 3 to 10.

Figure 3. Top 10 GO terms with the highest frequency identified as "biological process" for 10 plant species.

Figure 4. Top 10 GO terms with the highest frequency identified as "cellular component" for 10 plant species.

Figure 5. Top 10 GO terms with the highest frequency identified as "molecular function" for 10 plant species.

Figure 6. Top 10 cellular component GO terms in the cliques of size 10.

Figure 7. Top 10 molecular function GO terms in the cliques of size 10.

Figure 8. Top 10 biological process GO terms in the cliques of size 10.

**
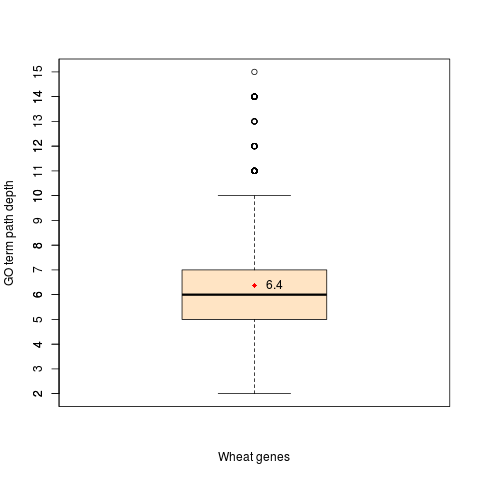
**

Figure 9. Box plot representing GO term path depth for wheat genes with newly assigned GO terms. The red dot represents the mean (6.4).


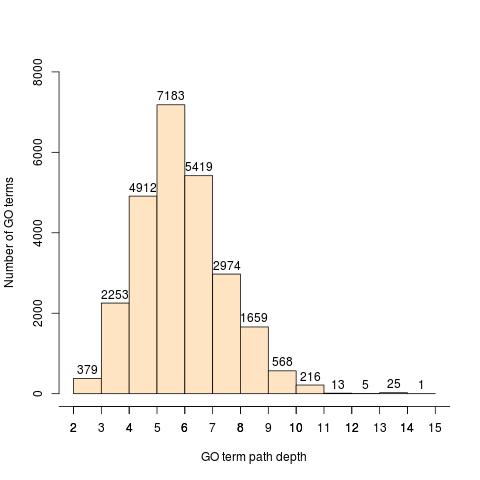


Figure 10. Histogram of GO term path depth for wheat genes with newly assigned GO terms.


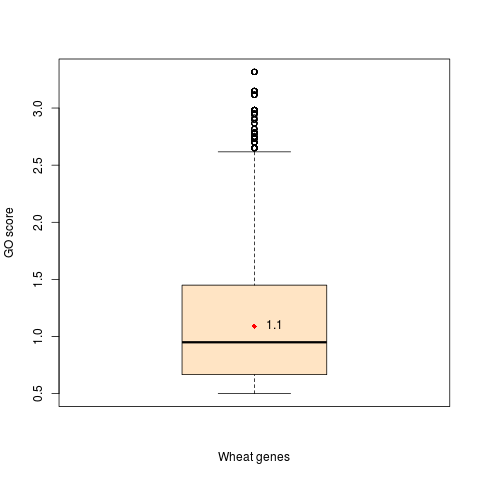


Figure 11. Box plot representing GO scores for wheat genes with newly assigned GO terms. The red dot represents the mean (1.1).


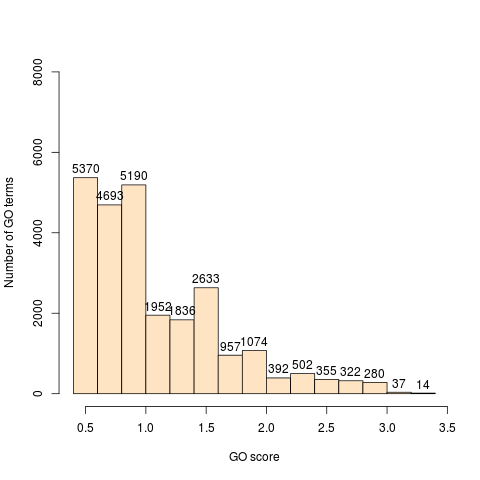


Figure 12. Histogram of GO scores for wheat genes with newly assigned GO terms.
